# Emergence of a New Pathogen: A Retrospective Study of HPeV-5 Central Nervous System Disease in Alberta, Canada

**DOI:** 10.3390/v16111684

**Published:** 2024-10-29

**Authors:** Grace George, Lea Restivo, Dolores Freire Jijon, Joan L. Robinson, Kevin Fonseca, Kanti Pabbaraju, Xiaoli Dong, Raymond Tellier, Tarah Lynch, Joseph V. Vayalumkal

**Affiliations:** 1 School of Public Health, University of Alberta, Edmonton, AB T6G 1C9, Canada; gageorge@ualberta.ca; 2 Department of Pediatrics, Alberta Children’s Hospital, University of Calgary, Calgary, AB T3B 6A8, Canada; leah.restivo@albertahealthservices.ca; 3 Department of Pediatrics, Stollery Children’s Hospital, University of Alberta, Edmonton, AB T6G 2B7, Canada; dolores.freirej@ug.edu.ec (D.F.J.); jr3@ualberta.ca (J.L.R.); 4 Alberta Precision Laboratories, Provincial Laboratory for Public Health, Calgary, AB T2N 4W4, Canada; kevin.fonseca@albertaprecisionlabs.ca (K.F.); kanti.pabbaraju@albertaprecisionlabs.ca (K.P.); xiaoli.dong@albertaprecisionlabs.ca (X.D.); tarah.lynch@albertaprecisionlabs.ca (T.L.); 5 Department of Microbiology Immunology and Infectious Diseases, University of Calgary, Calgary, AB T2N 4N1, Canada; 6 Department of Medicine, McGill University, Montreal, QC H4A 3J1, Canada; raymond.tellier@muhc.mcgill.ca; 7 Alberta Children’s Hospital Research Institute, Calgary, AB T2N 4N1, Canada

**Keywords:** human parechovirus, viral meningitis, encephalitis, meningo-encephalitis, pediatrics, recombinant

## Abstract

Human parechoviruses (HPeVs) are known to cause meningo-encephalitis among neonates and infants. We aimed to describe the epidemiology of HPeVs causing central nervous system infections in Alberta from 2014 to 2019 with comparison of known HPeV-3 and emerging HPeV-5. Genomic analysis was completed on a subset of HPeV-5 strains to understand genetic relatedness to other known strains. All cerebrospinal fluid (CSF) samples in Alberta with detection of HPeVs were identified and a case review of medical records was conducted, retrospectively, to gather demographic and clinical details. Descriptive and analytic statistics were used to describe and compare the characteristics of cases affected by HPeV-3 with HPeV-5. Genome amplification was completed on six HPeV-5 samples. During the study period, 18,882 CSF samples were tested; 56 were positive for HPeV-3 or HPeV-5, and 52 patients were included in this study (40 HPeV-3 cases and 12 HPeV-5). A total of 40% of cases occurred in 2016, and 64% of infections occurred in the months of August to October. The mean age of cases was 18 days for HPeV-5 compared with 26 days for HPeV-3 (*p* = 0.045). Phylogenetic comparison showed similarity to a recombinant strain reported in Australia. HPeV meningo-encephalitis affected neonates/infants, mostly in late summer/early fall, and genomic sequencing of new strains can contribute to understanding the epidemiology of HPeV infections globally.

## 1. Introduction

Human parechoviruses (HPeVs), first isolated in 1956, were initially classified as echovirus 22 and 23 serotypes of enteroviruses [1]. However, their distinct serologic and sequence divergence led to their reclassification into a new genus of *Parechovirus* in 1997 within the family *Picornaviridae* [1]. This genus is divided into four species, A to D [2]. Within Species A, the International Committee on Taxonomy of Viruses (ICTV) recognizes 18 types of HpeVs [3,4]. Infections occur worldwide, with HPeV-1 being the most reported, followed by HPeV 3. In temperate climates, each of these types shows different seasonal incidences and cycles of infection [5].

HPeVs predominantly affect the gastrointestinal and respiratory tracts with transmission occurring via the fecal–oral and respiratory routes [1,6]. The spectrum of disease ranges from asymptomatic infection or mild gastrointestinal or respiratory symptoms to infant sepsis, meningitis, encephalitis, or acute flaccid paralysis. Children under the age of 2 years are more likely to be symptomatic, whereas those under 6 months of age develop the most severe disease, possibly because they have higher rates of HPeV-3 infection than older children [7]. There are no known effective treatments for HPeVs.

HPeVs are increasingly recognized as important causes of central nervous system (CNS) infection in children, presumably because molecular testing has become more widely available. These infections occur almost exclusively in neonates and young infants. HPeV-3 causes most CNS infections [7,8], although HPeV-1, HPeV-4, HPeV-5, and HPeV-6 have occasionally been implicated. The disease is often severe, requiring intensive care unit (ICU) admission, with brain imaging sometimes showing white matter lesions and intracranial hemorrhage [7].

The first objective of this study was to describe the epidemiology of CNS disease due to HPeV-3 and HPeV-5 in Alberta from 2014 to 2019. Given the rarity of previous reports of CNS disease due to HPeV-5, a second objective was to describe the clinical spectrum and risk factors for CNS disease due to HPeV-5 and to compare the clinical features to HPeV-3 cases. Furthermore, we aimed to compare the genome sequence of the HPeV-5 positive samples with other HPeV-5 strains described in the literature.

## 2. Materials and Methods

All CSF samples in Alberta with detection of HPeVs (HPeV-3 or HPeV-5) from 2014 through 2019 were identified from the ProvLab database, which is the only laboratory in the province that tests clinical samples for viral etiologies. In Alberta, an enterovirus/parechovirus multiplex assay [9] was introduced in 2012 and performed as part of a panel on all cerebrospinal fluid (CSF) samples submitted for investigation of viral meningitis/encephalitis to the Alberta Precision Labs, Provincial Laboratory for Public Health of Alberta (ProvLab). Genotyping was performed using the capsid gene (in-house designed primers and protocol) on all HPeV-positive specimens identified in CSF from patients less than one year of age starting August 2014.

Genotyping was performed by Sanger sequencing of the capsid gene using in-house-designed primers and protocol where the viral load determines if the Sanger sequencing was successful. Representative HPeV-5-positive samples with the highest viral load were subjected to genome sequencing in order to understand if these were recombinants with genome segments from more virulent genotypes such as HPeV-3.

Electronic records and paper charts were reviewed for cases of all ages (including adults) to gather demographic and clinical details. Cases were excluded only if their medical records could not be obtained, typically because they were admitted to hospitals outside of Edmonton or Calgary, the two major tertiary care centers in the province.

To ensure data collection was standardized, four charts were reviewed initially, and any differences were rectified by the creation of a coding manual that provided a clear set of protocols and guidelines to instruct the reviewers in the collection of data.

Data collected included age, sex, symptoms, days of illness prior to presentation, past medical history, presence of suspected epidemiological risk factors (such as presence of sick contacts), month of the year, physical examination findings, laboratory results, imaging findings, need for ICU admission, complications, and duration of hospitalization. Neurological or developmental deficits noted at the time of discharge were recorded. Study data were collected and managed using Research Electronic Data Capture (REDCap Version 9.5x), a secure, web-based software platform designed to support data capture for research studies hosted at the University of Calgary [10,11]. This study received approval from the Conjoint Health Research Ethics Board (CHREB) at the University of Calgary (Certification #REB19-0274).

Descriptive statistics were used to identify and compare the spectrum of CNS disease caused by HPeV-5 to HPeV-3 and the potential risk factors for CNS disease caused by HPeV-5 and HPeV-3. Categorical variables were described by the distribution of HPeV-5 and HPeV-3 patients in each category as well as the number and percentage of patients. Scalar variables were described by the mean value and range for HPeV-5 and HPeV-3 patients. Analytical statistics were used to determine if there was a statistical difference between the distribution and mean values for HPeV-5 and HPeV-3 patients. For categorical variables, a chi-square test for independence was used to compare the distribution of the variables between HPeV-5 and HPeV-3 patients and determine if a statistical difference existed, testing the null hypothesis that the distribution of cases was similar between the two groups. If the distribution of cases for a variable was insufficient in either group, then no measure of association was computed. For scalar variables, a t-test was used to determine if there was a significant difference between the means of the two groups, testing the null hypothesis that the means in the two groups were similar. Before a t-test was completed, the assumptions for normality and homogenous variance were tested. A Shapiro–Wilks test was used to determine if the data followed a normal distribution and a Levene test was used to determine if the samples had equal variance. If the data failed to meet these assumptions, a Mann–Whitney test was used to determine if the distributions of both populations were equal.

In order to understand the phylogenetic and molecular characteristics of the complete genome for the HPeV-5 positives detected in Calgary in 2018, six samples were subjected to complete genome amplification to generate ten overlapping PCR fragments using a set of previously published primers [12]. RT-PCR was performed using the SuperScript™ III One-Step RT-PCR System with Platinum™ Taq High Fidelity DNA Polymerase (ThermoFisher, Waltham, Massachusetts, USA) using the primers at a final concentration of 0.6 mM. The reverse transcription was performed for 30 min at 55 °C followed by enzyme inactivation at 94 °C for 2 min. Amplification was performed using two steps. The first step included five cycles of denaturation, annealing, and extension at 94 °C for 30 s, 45 °C for 1 min, and 68 °C for 2 min, respectively. This was followed by 40 cycles of denaturation, annealing, and extension at 94 °C for 30 s, 50 or 55 °C (depending on the primer set) for 1 min, and 68 °C for 2 min, respectively. Nested PCR was performed with Platinum Taq II (ThermoFisher, Waltham, Massachusetts, USA) using a final concentration of 0.6 mM for the primers and 35 cycles of denaturation, annealing, and extension at 94 °C for 30 s, 50 or 55 °C (depending on the primer set) for 1 min and 68 °C for 2 min, respectively. Products were visualized by gel electrophoresis.

All available PCR products for each of the specimens were pooled and library prep was performed using the Oxford nanopore Ligation kit and sequenced on the MinION MK1B (Oxford Nanopore Technologies, Oxford, UK) with FLO-MIN114 flow cell. Base calling was performed using the high-accuracy model in real-time using Dorado (MinKNOW 24.02.16). Reads with dual barcodes were adapter-trimmed with Porechop 0.2.4, length- and quality-filtered using Chopper 0.6.0, and the host reads were removed using Hostile 0.1.0. These reads were mapped to an in-house reference database (HPeV-genomedb) including HPeV1–18 prototype strains from picornaviridae.com and the recombinant HPeV-5 clinical strain G001-19 [13] using MiniMap2 2.21; bcftools 1.20 was used to generate the HPeV draft whole-genome consensus sequences for each of the six patient specimens and positive control. Mafft (v7.505) alignments including the five closest hits for each sequence from HPeV-genomedb using BLAST and closely related sequences from NCBI were used to build phylogenetic trees for the VP1, 2C, and 3D genes using RaxML-NG (v1.2.2). A similar strategy was used to build whole genome trees using sequences longer than 1000 bp where the whole-genome multiple sequence alignment was filtered by removing columns in which more than 4 of the 7 sequences did not have a nucleotide at that position.

## 3. Results

### 3.1. Identifying Human Parechovirus Central Nervous System Infections

In Alberta, from 2014 to 2019, a total of 18,882 CSF samples were tested for HPeVs.

### 3.2. Characteristics of Patients

There were 56 patients identified with HPeV-3 or HPeV-5 CNS infection from 2014 to 2019. Four were excluded as medical records could not be obtained. A total of 52 patients (31 male) were included in this study. HPeV-3 was detected in 40 cases and HPeV-5 in 12 cases (Table 1). The mean age was 24 days (range 6–75 days). Two cases were preterm infants.

All were hospitalized (20 in Edmonton, 26 in Calgary, while 6 were admitted to smaller centers and never transferred). The mean length of stay was 5.1 days (range 2–27 days; median 4 days). Intensive care unit (neonatal or pediatric intensive care) admission was required for 16 patients (31%) for a mean length of stay of 3.7 days (range 1 to 8 days—data missing for two patients total). Four (8%) required mechanical ventilation. Two patients required vasopressor support. For 15 of the 16 patients admitted to intensive care, the average length of total hospitalization was 6.5 days (range 3–11 days—data missing for one patient). For the 36 patients not admitted to the PICU, the average length of hospitalization was 4.6 days (2–27 days). At presentation, seizures were reported in two patients. One had a brief absence seizure of less than a minute duration and the other had a focal seizure which became generalized. Four patients required TPN, and five required feeding via nasogastric tube. Ten patients (19%) had possible/confirmed necrotizing enterocolitis (NEC). Intravenous antibiotic use was documented for 50 patients (96%) for a mean duration of 2.9 days (data missing for 4 patients). No deaths were recorded in the 50 patients where data were available.

### 3.3. Coinfections

Nasopharyngeal swabs collected on 8 (*n* = 7 HPeV-3, *n* = 1 HPeV-5) patients revealed enterovirus or rhinovirus infection and one other patient (HPeV-3) was positive for Respiratory Syncytial Virus (RSV). Urine cultures had the growth of an organism in 10 patients (*n* = 8 HPeV-3, *n* = 2 HPeV-5). Pure growth of *Escherichia coli* was identified in 4 urine cultures, Whereas pure growth of *Enterococcus faecalis* was identified in 2 urine cultures; mixed growth of *E. coli* and *E.faecalis* was noted in 2 urine cultures. Other organisms noted from urine cultures included *Klebsiella oxytoca* in 1 patient and Coagulase negative Staphylococcus in 1 patient.

### 3.4. Magnetic Resonance Imaging Findings

Of the 10 patients who underwent magnetic resonance imaging (MRI), 4 had abnormal findings. Three cases with abnormal MRI were HPeV-3 cases. One had a small focus of diffusion restriction seen in the right anterior frontal lobe. Another had a sunburst pattern of multifocal areas of restricted diffusion in the supratentorial white matter consistent with encephalitis. The third had small focal areas of diffusion restriction in the deep white matter of the right frontal lobe, left parietal, and left temporal lobes with evidence of diffusion restriction in the splenium of the corpus callosum (reported as likely representing encephalitis). Mild irregularity of the M1 segment of the middle cerebral arteries bilaterally was reported to be likely related to vasculitis. The fourth case with an abnormal MRI was an HPeV-5 case. The MRI for this patient showed subependymal hemorrhage of the right lateral ventricle, and some white matter injuries in the right cerebral hemisphere appearing to be associated with small hemorrhages.

### 3.5. Outcomes

At the time of discharge, three patients had neurologic sequelae, including epilepsy (N = 1), focal neurological deficit (N = 1), and hypotonia (N = 1).

### 3.6. Timing of Infections

**Figure 1:**
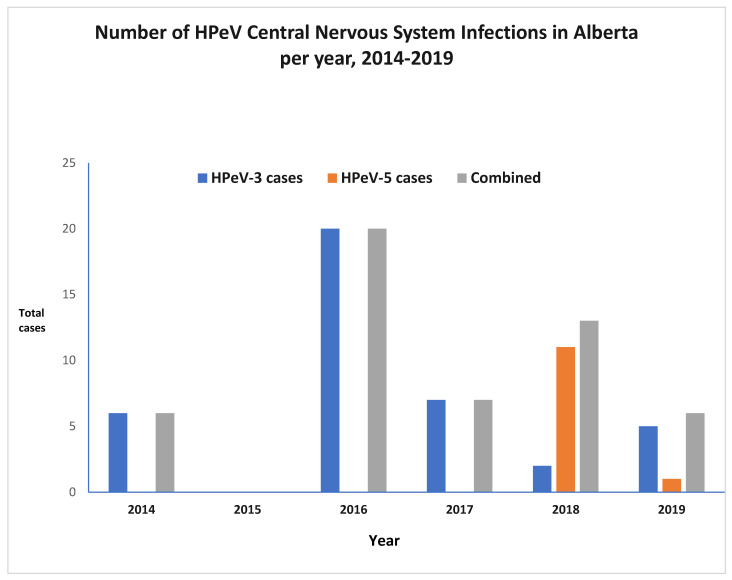
Distribution of HPeV CNS infection cases 2014–2019.

**Figure 2:**
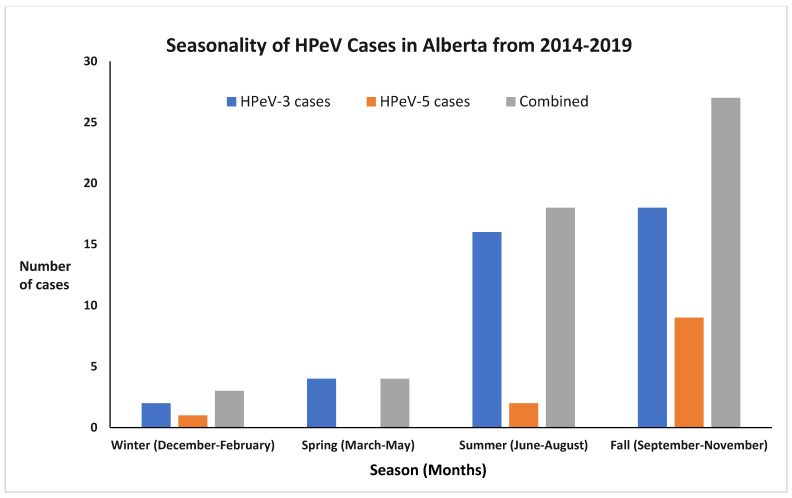
Seasonal distribution of HPeV CNS infection cases 2014–2019.

Forty percent of cases (*n* = 21) occurred in one year (2016) of this six-year study (Figure 1). The pattern of infections showed seasonality, with most infections occurring in the summer or fall seasons. There appeared to be a peak in August, September, and October in most years, with 33 infections (64%) occurring in these months. September was the most common month for infection with a total of 15 cases, followed by August with 11 cases (Figure 2).

### 3.7. Comparison Between Children Infected with HPeV-3 and HPeV-5

Patients differed only by age of presentation, 18 days in the HPeV-5 infections versus 26 days in the HPeV-3 infections (*p* = 0.045) (Table 1).

### 3.8. Genome Characterization of the HPeV-5-Positive Samples

**Figure 3:**
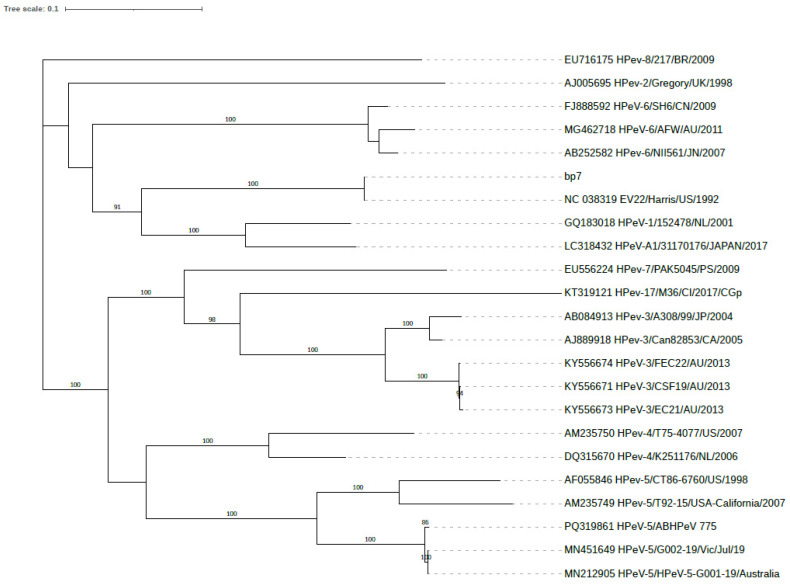
Maximum likelihood phylogenetic tree by RAxML (GTR + G model) based on HPeV whole-genome sequences. The phylogenetic tree includes a representative HPeV-5-positive sample from Alberta (PQ319861_HPeV-5/ABHPeV_775), a positive control sample HPeV-1_L02971 (bp7), and 21 publicly available HPeV whole-genome sequences from HPeV-genomedb. The Alberta sample (PQ319861_HPeV-5/ABHPeV_775) shares the most recent common ancestor with the recombinant HPeV-5 samples from Australia (13). The positive control sample (bp7-HPeV-1_L02971) is most closely related to other publicly available HPeV-1 virus genomes as expected. The bootstrap values greater than 80 were displayed on the branches. The scale bar corresponds to the expected mean number of nucleotide substitutions per site.

The sequenced samples had low viral loads, and the complete genome could not be generated for any of the samples. However, partial genome coverage for the six samples ranged from 2778 bp to 6829 bp, corresponding to 38.16% to 92.91% genome coverage. Phylogenetic comparison to HPeV-genomedb shows that the partial genomes clustered with G001-19 (Figure 3), and the VP1, 2C, and 3D gene sequences were most like HPeV5, HPeV1, and HPeV3, respectively, similar to the recombinant strain G001-19 reported in Australia.

Furthermore, these strains (all) show the presence of the arginine–glycine–aspartic acid (RGD integrin-binding motif) in the VP1 region [14], the absence of which is associated with more severe disease in infants.

## 4. Discussion

Our data reveal the emergence of HPeV-5 in Alberta in 2018 and its displacement of HPeV-3, followed in 2019 by a reemergence of HPeV-3. As in previous reports, HPeV-3 was the cause of most CNS infections of neonates. In recent years, HPeVs have been increasingly reported as important pathogens, which may be partly related to the availability of diagnostic testing. As in other published studies, a seasonal pattern was noted, with infections peaking in late summer and early fall. It is also noteworthy that the oldest case was 75 days old, highlighting the predisposition of young infants to this infection.

The recent emergence of HPeV-5 has been reported in other countries. Chamings et al. reported that 7 of 10 infants with HPeV infection in Southeastern Australia, from January to July 2019, had HPeV-5 [13]. All HPeV5-positive cases were under the age of 3 months and admitted to the hospital with fever, rash, lethargy, and/or sepsis-like clinical signs. In previous literature, HPeV5 was not typically associated with severe clinical signs, but the authors postulated increased severity through the acquisition of genes by HPeV5 from a more virulent HPeV and the absence of the RGD motif [13]. This fits with the fact that in Sapporo, Japan, seven clinical samples from national epidemiological surveillance data were positive for HPeV-5 in the summer of 2018 [15]; whole genome sequencing demonstrated that the virus was a recombinant of HPeV-3 and HPeV-5. A study from Ohio, USA reported only 1 case of human parechovirus type 5 (from a non-sterile site) out of 130 infants with HPeV detected from 2014 to 2016 [16]. This shows that HPeV-5 was present in North America two or more years prior to our study period.

The presence of the RGD motif, noted in our strains and those from Australia and Sapporo, Japan, has been previously hypothesized to be associated with less severe infections, as the virus can infect a wider range of cells utilizing other receptors [13,15]. However, the HPeV 5 strains from patients in our study and from Australia showed a significant degree of severity as our patients had neurologic infections requiring ICU support, of which three cases continued to show neurologic sequelae at discharge. Consequently, the absence of the RGD motif does not appear to play a significant role in determining the severity of disease. Representative HPeV-positive samples have been genotyped in Alberta since 2014 by sequencing the capsid gene. An HPeV-5-positive case was first detected in 2018, and genome sequencing shows the closest match to the recombinant strain reported in Australia. It has been speculated that the enhanced pathogenicity of this recombinant and other HPeV-4 could be due to the acquisition of the polymerase gene similar to that of HPeV-3, resulting in more efficient replication and thus increased severity of symptoms [13,17,18]. This recombinant strain has been reported in Europe; however, it is difficult to speculate where the viruses had the opportunity to recombine and the progenitor strain first appeared.

The only significant difference in the presentation of HPeV-3 and HPeV-5 cases in our study was the mean age of presentation; 18 days in the HPeV-5 infections vs. 26 days in the HPeV-3 infections (*p* = 0.045).

This fits with the Australian study, where 7 HPeV-5 infections were identified with an age range of 14–68 days, much younger than the 3 HPeV-1 cases they found with an age range of 267–615 days. [13]

In the previously mentioned large Ohio study, 1475 young febrile infants were evaluated from 2013 to 2016 at various body sites, and 130 (9%) were positive for HPeV in 1 or more body sites. It was noted that 53 (41%) were positive in CSF samples, but only 5 infants were positive in CSF alone. HPeV-3 was the most common type and was the only type detected in the CSF [16]. HPeV-5 was only noted in 1 patient. HPeV infections were detected year-round, peaked during late summer to early fall, and did not have a striking biennial pattern. In addition to fever and fussiness/irritability, infants commonly presented with rash, upper respiratory tract symptoms, diarrhea, and abdominal distension [16].

As in other studies [16,19], pleocytosis was not a common feature of the HPeV CNS infection in infants, occurring in only six patients (12%).

A diagnosis of possible necrotizing enterocolitis (NEC) in about 20% of our patients is notable and has been described in cases of HPeV viral sepsis beyond the neonatal period and in premature neonates. [19,20,21,22]. Other reports of HPeV infection in neonates and infants have noted findings suggestive of NEC, including abdominal distension, bloody stools, pneumatosis intestinalis, and even surgical abdomen [23,24]. It is not clear whether abdominal distension alone is a feature of HPeV infection or whether some infants truly have NEC.

In our study, 4 of 10 patients had abnormal MRI findings. These imaging abnormalities were consistent with the findings of other studies. In a recently published systematic review, various brain MRI abnormalities were described in association with HPeV CNS infections [25]. The authors described common findings in reviewed studies, which included restricted diffusion in deep white matter and periventricular white matter involving mainly the frontal zones [25]. They also noted involvement of parietal and temporal lobes, corpus callosum, and thalamus in some reports [25].

A case series that included 5 neonates with HPeV CNS infection noted specific white matter changes involving the periventricular and subcortical white matter [26]. They noted relative sparing of subcortical white matter, thalamus, basal ganglia, and infratentorial regions [26]. Another case series involving 6 infants reported the most characteristic finding was diffuse/multifocal frontal-predominant subcortical white matter and callosal involvement with associated low diffusivity [27]. They also reported that no cases exhibited signal abnormality within the basal ganglia, hippocampi, brain stem, and cerebellum. In a report from Philadelphia, 3 cases of parechovirus encephalitis imaging findings were highlighted; the two neonatal cases showed distinctive MRI brain patterns of injury including the characteristic radiating sunburst pattern of restricted diffusion in association with corpus callosum signal abnormality and bilateral swollen thalami [28]. In a study from Ireland, one case of HPeV meningo-encephalitis showed frontoparietal white matter changes on brain MRI [29]. Our cases showed various imaging abnormalities including diffusion restriction seen in the right anterior frontal lobe, sunburst pattern of multifocal areas of restricted diffusion in the supratentorial white matter, and other white matter injuries like other studies.

The strength of this study is that it is population-based and includes all cases of laboratory-confirmed HPeV CNS infections in all age groups in Alberta over a 6-year period. Furthermore, the number of cases of HPeV-5 is much larger than described in earlier studies, thus providing significantly more information on the epidemiology, genetics of the virus, clinical manifestations, and outcomes. It also provides detailed genomic analysis providing clues to the spread and genetic relatedness of HPeV in different parts of the world. One limitation is that it was a retrospective chart review conducted in only one Canadian province. CSF may not have been collected and tested if the patient’s presentation was mild or if the signs of CNS infection were subtle, which may have led to incomplete case findings. It is also possible that viral studies were not requested on the CSF samples of all patients. Furthermore, false-negative testing may have been an issue if testing was conducted very early in the course of illness and not repeated. Also, Neurodevelopmental outcomes were not ascertained following hospital discharge

## 5. Conclusions

Overall, our study adds to the body of knowledge on HPeVs in infants and provides the first Canadian data on the emergence of HPeV-5 as a pathogen in young infants. It appears that HPeV5 may have evolved and is now causing more CNS disease than in the past. Given the morbidity that can arise, it would be of great value to have therapy for HPeV. Typing of HPeV isolates can provide additional information on emerging trends in the epidemiology of this infection. HPeV-5 or other types may become more prominent in the future and could present unique clinical features that have not been described with other types. Typing may also provide insights into the future prognosis for the neurodevelopment of infants with CNS HPeV infection. Complete genome sequencing in addition to the standard practice of sequencing the capsid genes can contribute to the understanding of epidemiology, pathogenicity, and rate of recombination for HPeV.

## Figures and Tables

**Table 1:** Characteristics of cases (N = 52) and comparison of HPeV-3 and HPeV-5.

Variable	Total (N = 52)	HPeV-3 (N = 40)	HPeV-5 (N = 12)	*p*-Value
Age (in days) Median (range)	24 (6–75)	26 (6–75)	18 (7–45)	0.045
Male	31 (60%)	21 (53%)	10 (83%)	0.93
Hospital length of stay in days Mean (range)	5.1 (2–27)	5.0 (2–12)	5.8 * (2–27)	0.207
Known sick contact	21 (40%)	16 (40%)	5 (41%)	
ICU admission	16 (31%)	12 (30%)	4 (33%)	0.331
ICU length of stay in days (14 patients) Mean (range)	3.7 (1–8)	4 * (1–8)	2.7 * (2–3)	
Hospital length of stay for children admitted to ICU in days Mean (range)	6.5 (3–11)	6.7 (3–11)	6 * (3–10)	0.633
Hospital length of stay for children not admitted to ICU in days Mean (range)	4.6 (2–27)	4.3 (2–12)	5.8 (2–27)	0.236
Required nutritional support (TPN or NG feeds)	9 (17%)	8 (20%)	1 (8%)	0.231
Seizure	2 (4%)	1 (3%)	1 (8%)	0.139
Necrotizing enterocolitis (possible or confirmed)	10 (19%)	10 (25%)	0	0.092
Late preterm ***	2 (4%)	2 (3%)	0	
MRI performed	N = 10, 4 abnormal	N = 8, 3 abnormal	N = 2, 1 abnormal	0.923
Neurologic sequelae evident at discharge	3 (6%)	2 (5%)	1 (8%)	
Laboratory abnormalities				
Leukopenia	19 (37%)	16 (40%)	3 (25%)	0.499
Neutropenia	8 (15%)	6 (15%_	2 (17%)	1.0
Lymphopenia	25 (48%)	21 (53%)	4 (33%)	0.329
Thrombocytopenia	2 (4%)	2 (3%)		1.0
CSF sampled	N = 47 (90%)	N = 36 (90%)	N = 11 (92%)	
Elevated WBC	6 (12%)	5 (13%)	1 (8%)	0.457
Elevated protein	12 (24%)	9 (23%)	3 (25%)	0.974
Elevated CRP	3 (6%)	1 (3%)	2 (17%)	0.129
Hyponatremia	6 (12%)	5 (13%)	1 (8%)	1.0
Hyperkalemia	5 (10%)	3 (8%)	2 (17%)	0.329
Elevated transaminases	12 (24%)	10 (25%)	2 (17%)	0.709

* Data missing for 1 patient, *** 34–36 weeks gestational age. Abbreviations: CSF—cerebrospinal fluid; CRP—C-reactive protein; NG—nasogastric; ICU—intensive care unit; TPN—total parenteral nutrition; WBC—white blood cell count.

## Data Availability

The genomic data are openly available; high-quality consensus genomes were submitted to GenBank. http://www.ncbi.nlm.nih.gov/genbank (accessed on 9 October 2024). Study ID: GenBank Accession; ABHPeV_038: PQ319859; ABHPeV_972: PQ319860; ABHPeV_775: PQ319861. The original contributions presented in this study are included in the article, further inquiries can be directed to the corresponding author.
